# PML-not a surprise in four young HIV infected patients

**DOI:** 10.1186/1471-2334-14-S4-P34

**Published:** 2014-05-29

**Authors:** Andreea Cioară, Cristian Marcu, Roxana Iubu, Daniela Marcu, Ariana Almaşu, Mirela Flonta, Mihaela Lupşe, Corina Itu

**Affiliations:** 1 Infectious Disease Department, Iuliu Hațieganu University of Medicine and Pharmacy, Cluj-Napoca, Romania; 2 Infectious Diseased Clinic, Cluj-Napoca, Romania

## 

An important cause of mortality in patients from Romanian cohort remains progressive multifocal leukoencephalopathy (PML) caused by the polyomavirus, JC virus (JCV).

PML is an opportunistic infection and one of the AIDS-defining conditions in HIV-infected patients and is associated with both HIV-1 and HIV-2.

We perform a retrospective study in which we included 4 patients from Cluj-Napoca HIV Center. These patients belong to Romanian cohort and were with poor adherence to treatment.

We collected information on clinical and immunological status, microbiologic and virological analysis, neuroimaging, treatment and outcome.

From 4 patients with a median age of 20.75 years at the moment of PML diagnosis, 3 were male and 1 was a female, 3 died and 1 is alive.

All patients were already in stage AIDS C3 at the moment of PML diagnosis and with a CD4 lymphocytes level below 20 cells/cmm.

The onset was insidious in all cases with focal neurological signs (hemiparesis, facial palsy, dysarthria and mental alteration) without fever.

In two cases we performed lumbar puncture that revealed clear CSF, with normal level of glucose and proteins, negative Gram stain and negative culture but with JC virus load positive (RT-PCR JCV from CSF).

The imagistic methods (cerebral MRI) showed in all cases lesions localized in white matter, without mass effect from these lesions. For two cases we completed examination with cerebral MR spectroscopy which revealed spectroscopic disorders specific for PML.

In all cases we excluded other etiologies by serological, bacteriological and imagistic tests.

All patients received cART with good penetration in CSF and corticotherapy, but in 3 cases neurologic disorders evolved to tetraparesis, aphasia and profound altered mental status and finally death.

1 patient had a good neurologic recovery and remains with easy ataxia and dysarthria.

In these cases PML is a result of low adherence to treatment of patients from Romanian cohort and opportunistic infection was not a surprise. The most important differential diagnosis was HIV encephalopathy.

We can conclude that our patients with PML had a poor prognosis despite intensive cART with good penetration in CSF.

